# Identification of Five Driver Gene Mutations in Patients with Treatment-Naïve Lung Adenocarcinoma in Taiwan

**DOI:** 10.1371/journal.pone.0120852

**Published:** 2015-03-19

**Authors:** Kuo-Hsuan Hsu, Chao-Chi Ho, Te-Chun Hsia, Jeng-Sen Tseng, Kang-Yi Su, Ming-Fang Wu, Kuo-Liang Chiu, Tsung-Ying Yang, Kun-Chieh Chen, Hean Ooi, Tzu-Chin Wu, Hung-Jen Chen, Hsuan-Yu Chen, Chi-Sheng Chang, Chung-Ping Hsu, Jiun-Yi Hsia, Cheng-Yen Chuang, Chin-Hung Lin, Jeremy J. W. Chen, Kuan-Yu Chen, Wei-Yu Liao, Jin-Yuan Shih, Sung-Liang Yu, Chong-Jen Yu, Pan-Chyr Yang, Gee-Chen Chang

**Affiliations:** 1 Division of Critical Care and Respiratory Therapy, Department of Internal Medicine, Taichung Veterans General Hospital, Taichung, Taiwan; 2 Institute of Biomedical Sciences, National Chung Hsing University, Taichung, Taiwan; 3 Division of Pulmonary Medicine, Department of Internal Medicine, National Taiwan University Hospital and National Taiwan University College of Medicine, Taipei, Taiwan; 4 Department of Internal Medicine, China Medical University Hospital, Taichung, Taiwan; 5 Department of Respiratory Therapy, China Medical University, Taichung, Taiwan; 6 Division of Chest Medicine, Department of Internal Medicine, Taichung Veterans General Hospital, Taichung, Taiwan; 7 Department of Clinical Laboratory Sciences and Medical Biotechnology, College of Medicine, National Taiwan University, Taipei, Taiwan; 8 Department of Laboratory Medicine, National Taiwan University Hospital, Taipei, Taiwan; 9 Divisions of Medical Oncology and Pulmonary Medicine, Department of Internal Medicine, Chung Shan Medical University Hospital, Taichung, Taiwan; 10 School of Medicine, Chung Shan Medical University, Taichung, Taiwan; 11 Division of Chest Medicine, Department of Internal Medicine, Taichung Tzu-Chi Hospital, Taichung, Taiwan; 12 School of Post-baccalaureate Chinese Medicine, Tzu Chi University, Hualien, Taiwan; 13 Faculty of Medicine, School of Medicine, National Yang-Ming University, Taipei, Taiwan; 14 Department of Critical Care Medicine, Taichung Tzu-Chi Hospital, Taichung, Taiwan; 15 Department of Medical Imaging and Radiological Sciences, Central Taiwan University of Science and Technology, Taichung, Taiwan; 16 Department of Medical Research, China Medical University Hospital, China Medical University, Taichung, Taiwan; 17 School of Medicine, Tzu Chi University, Hualien, Taiwan; 18 Division of Pulmonary Medicine, Chung Shan Medical University Hospital, Taichung, Taiwan; 19 Division of Pulmonary and Critical Care Medicine, China Medical University Hospital, Taichung, Taiwan; 20 Institute of Statistical Science, Academia Sinica, Taipei, Taiwan; 21 Division of Thoracic Surgery, Department of Surgery, Taichung Veterans General Hospital, Taichung, Taiwan; 22 School of Medicine, National Yang-Ming University, Taipei, Taiwan; 23 Graduate Institute of Clinical Medicine, College of Medicine, National Taiwan University, Taipei, Taiwan; 24 NTU Center for Genomic Medicine, National Taiwan University College of Medicine, Taipei, Taiwan; 25 Department of Pathology and Graduate Institute of Pathology, College of Medicine, National Taiwan University, Taipei, Taiwan; 26 Center for Optoelectronic Biomedicine, College of Medicine, National Taiwan University, Taipei, Taiwan; Seoul National University, KOREA, REPUBLIC OF

## Abstract

**Background:**

It is important to select appropriate targeted therapies for subgroups of patients with lung adenocarcinoma who have specific gene alterations.

**Methods:**

This prospective study was a multicenter project conducted in Taiwan for assessment of lung adenocarcinoma genetic tests. Five oncogenic drivers, including *EGFR*, *KRAS*, *BRAF*, *HER2* and *EML4-ALK* fusion mutations, were tested. *EGFR*, *KRAS*, *BRAF* and *HER2* mutations were assessed by MALDI-TOF MS (Cohort 1). *EML4-ALK* translocation was tested by Ventana method in *EGFR*-wild type patients (Cohort 2).

**Results:**

From August 2011 to November 2013, a total of 1772 patients with lung adenocarcinoma were enrolled. In Cohort 1 analysis, *EGFR*, *KRAS*, *HER2* and *BRAF* mutations were identified in 987 (55.7%), 93 (5.2%), 36 (2.0%) and 12 (0.7%) patients, respectively. Most of these mutations were mutually exclusive, except for co-mutations in seven patients (3 with *EGFR* + *KRAS*, 3 with *EGFR* + *HER2* and 1 with *KRAS* + *BRAF*). In Cohort 2 analysis, 29 of 295 *EGFR*-wild type patients (9.8%) were positive for *EML4-ALK* translocation. *EGFR* mutations were more common in female patients and non-smokers and *KRAS* mutations were more common in male patients and smokers. Gender and smoking status were not correlated significantly with *HER2*, *BRAF* and *EML4-ALK* mutations. *EML4-ALK* translocation was more common in patients with younger age.

**Conclusion:**

This was the first study in Taiwan to explore the incidence of five oncogenic drivers in patients with lung adenocarcinoma and the results could be valuable for physicians in consideration of targeted therapy and inclusion of clinical trials.

## Introduction

Lung cancer is the leading cause of cancer-related death worldwide [1], as well as in Taiwan. To improve the survival for advanced lung adenocarcinoma, strategies to target driver gene alterations are important and currently under intensive investigation.

Several molecular alterations are known to be involved in tumorigenesis of lung adenocarcinoma, such as *epidermal growth factor receptor (EGFR)*, *Kirsten rat sarcoma viral oncogene homolog (KRAS)*, *v-raf murine sarcoma viral oncogene homolog B (BRAF)*, *human epidermal growth factor receptor 2 (HER2)*, and *echinoderm microtubule-associated protein-like 4-anaplastic lymphoma kinase (EML4-ALK)* fusion mutations. Mutations in these genes are responsible for both the initiation and maintenance of lung adenocarcinoma [2]. By understanding the biological functions of these driver genes, it may be possible to develop specific therapies for lung cancer with known driver gene mutations.

Recently, Kris et al. demonstrated that patients with an oncogenic driver mutation who received the corresponding targeted therapy had a significantly longer survival time than those with a driver mutation who did not receive the targeted therapy and those without a driver mutation [3]. In comparison with chemotherapy, EGFR-tyrosine kinase inhibitors (TKI), including gefitinib, erlotinib and afatinib, have provided a better outcome and quality of life for patients with advanced *EGFR*-mutant non-small cell lung cancer (NSCLC) and have become one of the standard first line therapies [4–6]. Similar efficacy was observed in *EML4-ALK* positive lung cancer patients treated with crizotinib and second-generation ALK inhibitors [7,8].

Many drugs are available now and more will be developed for patients with specific driver gene mutations. Therefore, we decided to conduct a prospective study in five medical centers in Taiwan to explore the incidence of driver gene mutations and to define subgroups of patients in whom candidate driver gene alterations are enriched. Analyses of five driver genes, including *EGFR*, *KRAS*, *BRAF*, *HER2* and *EML4-ALK*, were performed in patients with treatment naïve lung adenocarcinoma.

## Materials and Methods

### Patients

This multicenter prospective observational study was conducted in five medical centers in Taiwan, including Taichung Veterans General Hospital, National Taiwan University Hospital, China Medical University Hospital, Chung Shan University Hospital and Taichung Tzu Chi Hospital. To be eligible for the study, patients were required to have treatment-naïve and pathologically confirmed lung adenocarcinoma and available tumor specimens for genetic analysis. Patients were excluded if they had lung cancer with histology other than adenocarcinoma, including those with carcinoma not otherwise specified (NOS), or other active malignancy. This study was approved by the Institutional Review Board of the participating institutes, including, Institutional Review Board of Taichung Veterans General Hospital (IRB No. C08197), National Taiwan University Hospital Research Ethics Committee (IRB No. 201111039RIC), Institutional Review Board of Chung Shan Medical University Hospital (IRB No. CS12022), China Medical University and Hospital Research Ethics Committee (IRB No. DMR100-IRB-284[CR-2]) and Taichung Tzu Chi Hospital Research Ethics Committee (IRB No. REC102–7). Written informed consents for genetic testing and clinical data records were obtained from all patients.

### Identification of driver mutations

Five oncogenic drivers, including *EGFR*, *KRAS*, *BRAF*, *HER2* and *EML4-ALK*, were tested. *EGFR*, *KRAS*, *BRAF* and *HER2* mutations were assessed by matrix-assisted laser desorption ionization-time of flight mass spectrometry (MALDI-TOF MS) since August 2011. *EML4-ALK* translocation was tested by Ventana method in patients with *EGFR*-wild type lung adenocarcinoma from July 2013. All the tests were performed by ISO15189-certified TR6 Pharmacogenomics Lab (PGL), National Research Program for Biopharmaceuticals (NRPB), the National Center of Excellence for Clinical Trial and Research of NTUH.

Tumor specimens were procured for *EGFR*, *KRAS*, *BRAF* and *HER2* mutation analyses as previously described [9]. Briefly, DNA was extracted from the tumors using a QIAmp DNA Mini kit (Qiagen, Valencia, CA) following the manufacturer’s protocol. The genetic alterations of *EGFR*, *KRAS*, *BRAF* and *HER2* were detected by MALDI-TOF MS based on the methods used in our previous reports with modifications [10,11]. Briefly, we expanded the multiplex gene-testing panel to include *EGFR*, *KRAS*, *BRAF* and *HER2* genes. We performed the analysis according to manufacturer’s protocol for MassARRAY system (Sequenom, San Diego, CA). In the biochemical reaction, polymerase chain reaction (PCR) followed by single nucleotide extension was performed by using primers and corresponding detection probes to amplify the region containing each target mutation. After SpectroClean Resin clean up, samples were loaded onto the matrix of SpectroCHIP by Nanodispenser (Matrix) and then analyzed by Bruker Autoflex MALDI-TOF MS. Data were collected and analyzed by Typer 4 software (Sequenom, San Diego, CA). The PCR primers and probes used in the present study were summarized in S1 Table.

Because *EGFR* mutations and *EML4-ALK* translocations are almost mutually exclusive [12], assessment of *EML4-ALK* translocations was only performed in patients with *EGFR*-wild type lung adenocarcinoma in this project by Ventana method [13]. Briefly, the assay (Ventana IHC, Ventana, Tucson, AZ) was a fully automated IHC assay developed by Ventana, using the pre-diluted Ventana anti-ALK (D5F3) Rabbit monoclonal primary antibody, together with the Optiview DAB IHC detection kit and Optiview Amplification kit on the Benchmark XT stainer. Each specimen was also stained with a matched Rabbit Monoclonal Negative Control Immunoglobulin antibody. The manufacture’s scoring algorithm was a binary scoring system (positive or negative for ALK status), which we adopted for evaluating the staining results. Neoplastic cells labeled with the ALK IHC assay were evaluated for presence or absence of the DAB signal. Presence of strong granular cytoplasmic staining in tumor cells (any percentage of positive tumor cells) was deemed to be ALK positive, while absence of strong granular cytoplasmic staining in tumor cells was deemed to be ALK negative.

### Data records and response evaluation

Activating mutation of *EGFR* gene is the most common genetic alteration in lung adenocarcinoma in East Asians [14]. Therefore, in addition to exploration of the incidence of driver mutations, we also evaluated the efficacy of EGFR-TKI in patients with *EGFR*-mutant lung adenocarcinoma. Clinical data for analysis included patients’ age, gender, Eastern Cooperative Oncology Group performance status (ECOG PS), tumor stage and smoking status. TNM (tumor, node, and metastases) staging was done according to the 7th edition of the American Joint Committee for Cancer (AJCC) staging system [15]. Chest computed tomographies (CT), including the liver and adrenal glands, and other required imaging studies for response evaluation were reviewed by two chest physicians. Unidimensional measurements as defined by Response Evaluation Criteria in Solid Tumors (RECIST) version 1.1 were used in this study [16].

### Statistical methods

Univariate analyses by Fisher’s exact test and Pearson Chi-square test were conducted on the frequency of five oncogenic drivers and on the objective response rate (ORR) and disease control rate (DCR) of EGFR-TKI therapy to evaluate the effects of clinical factors relating to patient and disease characteristics. Multivariate analyses of ORR and DCR were performed using logistic regression model. The Kaplan—Meier method was used to estimate progression-free survival (PFS) and overall survival (OS). Differences in survival time were analyzed using the log-rank test. Multivariate analyses of PFS and OS were performed using Cox proportional hazard model. All statistical tests were done with SPSS 15.0 (SPSS, Chicago, IL). Two-tailed tests and p values <0.05 for significance were used.

## Results

### Patient characteristics

From August 2011 to November 2013, a total of 1772 patients with treatment-naïve lung adenocarcinoma were enrolled as Cohort 1 for *EGFR*, *KRAS*, *BRAF* and *HER2* genetic analyses. With the development of Ventana method in July 2013 and the availability of tumor tissues, *EML4-ALK* translocation testing was performed in total 295 patients with *EGFR*-wild type lung adenocarcinoma, who were enrolled as Cohort 2. The baseline characteristics are shown in Table 1. Briefly, in Cohort 1, the median age was 58 years (range 21–100), 821 patients (46.3%) were male, 1179 patients (66.5%) were non-smokers and 1248 patients (70.4%) had advanced stage diseases (stage IIIb or IV). In Cohort 2, the median age was 61 yeas (range 21–100), 150 patients (50.8%) were male, 177 patients (60.0%) were non-smokers and 198 patients (67.1%) had advanced stage diseases.

**Table 1:** Characteristics and demographic data.

Characteristics	Cohort 1*	Cohort 2**
	(n = 1772)	(n = 295)
Age (years), median (range)	58 (21–100)	61 (21–100)
Gender		
Male, n (%)	821 (46.3)	150 (50.8)
Female, n (%)	951 (53.7)	145 (49.2)
Smoking status		
Non-smokers, n (%)	1179 (66.5)	177 (60.0)
Current/former smokers, n (%)	593 (33.5)	118 (40.0)
Stage, n (%)		
I	290 (16.4)	49 (16.6)
II	81 (4.6)	18 (6.1)
IIIa	141 (8.0)	29 (9.8)
IIIb	121 (6.8)	17 (5.8)
IV	1127 (63.6)	181 (61.4)
N/A	12 (0.6)	1 (0.3)

*Cohort 1: lung adenocarcinoma.

**Cohort 2: epidermal growth factor receptor-wild type lung adenocarcinoma.

N/A, not applicable.

### Incidence of five oncogenic drivers

**Fig 1:**
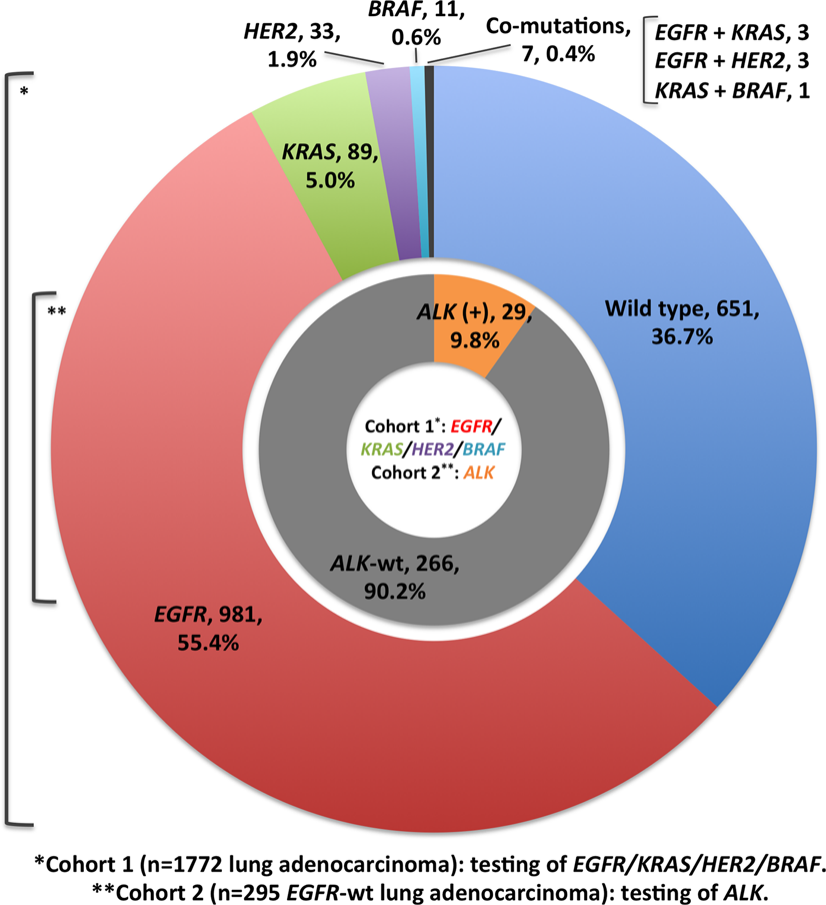
Spectrum of genetic alterations among patients with lung adenocarcinoma.

The oncogenic driver mutations detected in the present study are summarized in Table 2. The incidence of five driver gene mutations is shown in Fig. 1 and Table 2. In the Cohort 1 analysis, 987 of 1772 lung adenocarcinoma patients (55.7%) harbored activated *EGFR* mutations, which accounted for the majority of diver gene mutations. Mutations in *KRAS*, *HER2* and *BRAF* genes were identified in 93 (5.2%), 36 (2.0%) and 12 (0.7%) patients, respectively. A total of 651 patients (36.7%) had no detectable driver gene mutation. Majority of the genetic alterations were mutually exclusive, except for co-mutations in seven (0.4%) patients (3 with *EGFR* and *KRAS* mutations, 3 with *EGFR* and *HER2* mutations and 1 with *KRAS* and *BRAF* mutations). In the Cohort 2 analysis, 29 of 295 patients with *EGFR*-wild type lung adenocarcinoma (9.8%) were found to have *EML4-ALK* translocation and none of them harbored other driver gene mutations.

**Table 2:** Characteristics of genetic alterations assessed in the present study.

Gene*	Exon/Domain	Patient No.	Assessed genetic alterations
*EGFR*	Exon 18	18	E709A, E709G, E709V
			G719A, G719C, G719N, G719S
	Exon 19	434	E746-A750del, E746-T751>A,
			E746-S752>V, L747-A750>P, L747-T751>P,
			L747-S752del, L747-T751del, L747-P753>S,
			E746-T751>I, E746-T751del, E746-S752>A,
			E746-S752>D, L747-A750>P, L747-T751>Q,
			L747-E749del, L747-P753>Q, L747-T751>S
	Exon 20	3	T790M, S768I
	Exon 21	483	L858R, L858Q, L861Q
	Exon 18–21	49	Complex mutations
*KRAS*	Codon 12	89	G12S, G12R, G12C, G12D, G12A, G12V
	Codon 13	4	G13S, G13C, G13R, G13D, G13V, G13A
*HER2*	Exon 20	36	A775-G776insYVMA
*BRAF*	Exon 15	12	V600E
*ALK*	N/A	29	*EML4-ALK* translocation

EGFR, epidermal growth factor receptor; ALK, anaplastic lymphoma kinase; EML4, echinoderm microtubule-associated protein like 4.

*Cohort 1 (1772 patients with lung adenocarcinoma) testing of *EGFR*, *KRAS*, *HER2* and *BRAF* mutations and cohort 2 (295 patients with *EGFR*-wild type lung adenocarcinoma) testing of *EML4-ALK* translocation.

**Fig 2:**
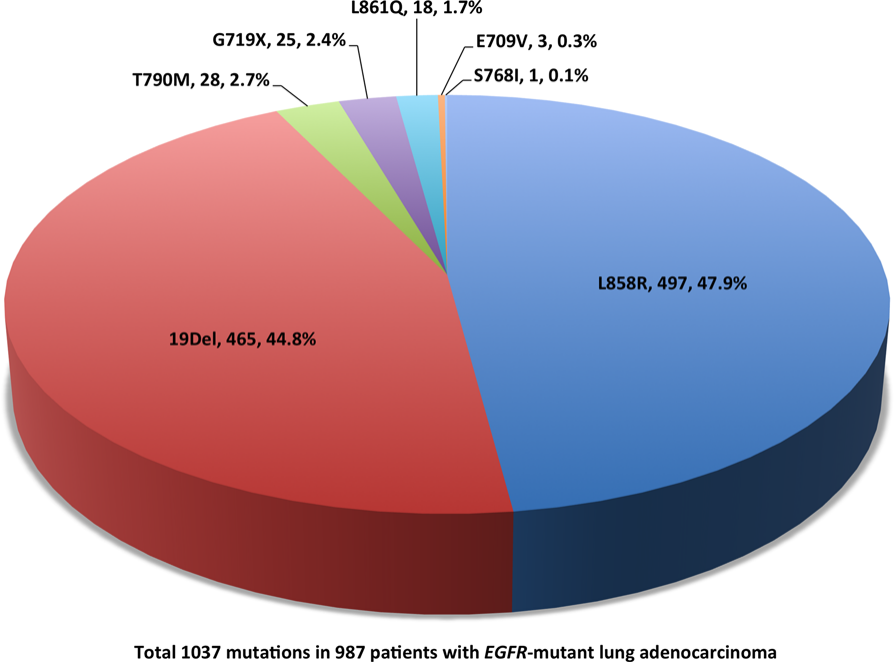
Epidermal growth factor receptor (EGFR) mutation spectrum among patients with EGFR-mutant lung adenocarcinoma (complex mutations are counted as separated mutation types here).

The detail of *EGFR* mutations is depicted in Fig. 2. A total of 1037 *EGFR* mutations were detected in 987 patients, including 49 patients (5.0%) who harbored complex mutations. Exon 19 in-frame deletions (44.8%) and exon 21 L858R (47.9%) were the major types of *EGFR* mutations. Furthermore, a total of 28 patients had primary exon 20 T790M mutation in the treatment-naïve tumor samples, mainly as part of complex mutations.

### Association between patient characteristics and driver gene mutations

The analysis of association between patient characteristics and driver gene mutations is shown in Table 3. For *EGFR* mutations, a significantly higher mutation rate was noted in female then in male patients (65.2 vs. 44.7%, P < 0.001) and in non-smokers then in smokers (63.9 vs. 39.5%, P < 0.001). Neither age nor tumor stage was significantly correlated with *EGFR* mutation.

**Table 3:** Univariate analysis of genetic alterations and clinical characteristics.

	*EGFR* *		*KRAS* *		*HER2* *		*BRAF* *		*ALK* **	
Characteristics	n (%)	P value	n (%)	P value	n (%)	P value	n (%)	P value	n (%)	P value
Age (years)		0.501		0.200		0.502		0.777		0.002
≤ 65	536 (55.0)		45 (4.6)		22 (2.3)		6 (0.6)		25 (14.0)	
> 65	451 (56.6)		48 (6.0)		14 (1.8)		6 (0.8)		4 (3.4)	
Gender		<0.001		<0.001		0.312		0.781		0.331
Male	367 (44.7)		76 (9.3)		20 (2.4)		5 (0.6)		12 (8.0)	
Female	620 (65.2)		17 (1.8)		16 (1.7)		7 (0.7)		17 (11.7)	
Smoking status		<0.001		<0.001		1.000		1.000		0.167
Non-smokers	753 (63.9)		20 (1.7)		24 (2.0)		8 (0.7)		21 (11.9)	
Current/former smokers	234 (39.5)		73 (12.3)		12 (2.0)		4 (0.7)		8 (6.8)	
Stage, n (%)^#^		0.187		1.000		0.015		0.754		0.837
I-IIIa	298 (58.2)		27 (5.3)		4 (0.8)		4 (0.9)		10 (10.4)	
IIIb-IV	682 (54.6)		66 (5.3)		32 (2.6)		8 (0.6)		19 (9.6)	

EGFR, epidermal growth factor receptor; ALK, anaplastic lymphoma kinase.

*Cohort 1 (1772 patients with lung adenocarcinoma) testing of *EGFR*, *KRAS*, *HER2* and *BRAF* mutations.

**Cohort 2 (295 patients with *EGFR*-wild type lung adenocarcinoma) testing of *EML4-ALK* translocation.

^#^Exclude 12 patients of cohort 1 and 1 patients of cohort 2 with incomplete data on tumor staging For *KRAS* mutations, a significantly higher mutation rate was noted in male then in female patients (9.3 vs. 1.8%, P < 0.001) and in smokers then in non-smokers (12.3 vs. 1.7%, P < 0.001). Neither age nor tumor stage were correlated with *KRAS* mutation. For *HER2* mutations, patients with more advanced diseases were associated with a higher mutation rate (2.6% in stage IIIb-IV vs. 0.8% in stage I-IIIa, P = 0.015). For *BRAF* mutation, there was no significant association between patient characteristics and the mutation rate.

In the Cohort 2 analysis, age was the only factor that correlated significantly with *EML4-ALK* translocation and patients with younger age (≤ 65 years) had a higher mutation rate than patients aged more than 65 years (14.0 vs. 3.4%, P = 0.002). Moreover, we observed a trend toward a higher mutation rate in non-smokers compared with smokers (11.9 vs. 6.8%) but the trend did not reach a level of significance (P = 0.167). Neither age nor tumor stage was correlated significantly with *EML4-ALK* translocation.

### Efficacy of EGFR-TKI in EGFR-mutant lung adenocarcinoma

In total, 352 patients who had *EGFR*-mutations and lesions that could be measured for treatment responses received EGFR-TKI therapy as the first line treatment (336 with gefitinib and 16 with erlotinib). The drug prescribed was chosen by the physicians in charge. Three patients achieved complete response, 203 patients achieved partial response, 95 patients had stable disease and 51 patients had progressive disease. The ORR and DCR were 58.5% and 85.5%, respectively. Univariate analyses of ORR and DCR are shown in Table 4. Patients with exon 19 deletions were associated with a higher ORR than those with L858R and other uncommon mutations (68.2 vs. 51.2 vs. 48.0%, P = 0.004). No other factors correlated significantly with ORR. In DCR analysis, females, non-smokers, patients with better ECOG PS (0–1) and those with exon 19 deletions were associated with a significantly higher DCR (P = 0.001, 0.013, <0.001 and 0.004, respectively). In multivariate logistic regression model, the exon 19 deletion was the only factor that predicted both a higher ORR (odds ratio 2.04, 95% CI 1.31–3.17, P = 0.002) and a higher DCR (odds ratio 2.40, 95% CI 1.21–4.77, P = 0.012). Gender and ECOG PS (0–1) remained significantly correlated with DCR in multivariate analysis (data not shown).

**Table 4:** Univariate analysis of objective response rate and disease control rate in patients harboring *EGFR* mutations (n = 352).

	Patient No.	ORR (%)	P value*	DCR (%)	P value*
Age (yrs)			0.449		0.880
≤ 65	189	56.6		85.2	
> 65	163	60.7		85.9	
Gender			0.147		0.001
Male	131	53.4		77.1	
Female	221	61.5		90.5	
Smoking			0.208		0.013
Non-smokers	266	60.5		88.3	
Current/former smokers	86	52.3		76.7	
ECOG PS^#^			0.660		<0.001
0–1	289	58.5		88.6	
≥ 2	57	54.4		68.4	
Stage			0.564		0.393
IIIb	12	50.0		75.0	
IV	340	58.8		85.9	
Mutation types			0.004		0.004
Exon 19 deletions	157	68.2		91.1	
Exon 21 L858R	170	51.2		82.9	
Others	25	48.0		68.0	
EGFR-TKIs			0.448		0.142
Gefitinib	336	58.0		84.8	
Erlotinib	16	68.8		100	

EGFR, epidermal growth factor receptor; ORR, objective response rate; DCR, disease control rate; ECOG PS, ECOG PS, Eastern Cooperative Oncology Group performance status.

*Mutation types by Pearson Chi-square test; otherwise by Fisher’s exact test.

^#^Exclude 6 cases with missing ECOG PS data.

The median PFS and OS were 11.6 (95% CI 10.0–13.2) and 39.4 (95% CI not applicable) months, respectively. In the univariate analysis, non-smokers and patients with ECOG PS 0–1 were associated with a significant longer PFS and ECOG PS (0–1) was the only factor that predicted a longer OS significantly (data not shown). Although patients harbored exon 19 deletions were associated with both a higher ORR and DCR, neither PFS (HR 0.74, 95% CI 0.55–1.00, P = 0.053) nor OS (HR 0.70, 95% CI 0.42–1.16, P = 0.163) was significantly correlated with *EGFR* mutation types in multivariate analysis. In multivariate Cox proportional hazard model, ECOG PS (0–1) was the only factor that independently predicted both a longer PFS (HR 0.67, 95% CI 0.46–0.97, P = 0.034) and OS (HR 0.46, 95% CI 0.27–0.79, P = 0.005).

## Discussion

The success of *EGFR* and *ALK* inhibitor therapies in lung cancer patients with *EGFR* mutations and *EML4-ALK* translocations initiated the era of targeted therapy in advanced NSCLC [5,8] and shifted treatment from platinum-based chemotherapy to molecularly targeted therapy. Recent genomic studies in lung adenocarcinoma have identified other potential therapeutic targets, such as mutations in *KRAS*, *HER2* and *BRAF*, *ROS1* rearrangements, *RET* fusions and *MET* amplification [17,18]. As the current trend of NSCLC treatment, especially lung adenocarcinoma, has been shifted from tumor stage-based to more individualized therapies based on histological and molecular characteristics [19], the increasing importance of genomic information should prompt physicians to obtain adequate tissue specimens when planning diagnostic procedures [20].

In East Asia, there are several unique characteristics of lung cancer [21], including the predominance of adenocarcinoma over other cell types and a large proportion of never smoker and female patients. Such features may provide keys to further investigate the treatment or prevention of lung cancer in East Asians. Moreover, the incidences of activating mutations of oncogenic drivers in Western and Asian populations are different, especially the *EGFR* and *KRAS* mutations [3,18,22,23]. In the present study, we showed that the incidence of *EGFR* mutations in Taiwanese lung adenocarcinoma patients was 55.7%, which was similar to the results of PIONEER study [14]. *EGFR* mutation is the most important biomarker for predicting the outcome of EGFR-TKI therapy. In the present study, the ORR of EGFR-TKI therapy was 58.5%, which was slightly lower than that of randomized controlled trials. However, we also enrolled patients with poor ECOG PS (2 or more) and outcome with regard to DCR, PFS and OS was similar to previous studies [4,5]. The incidence of *KRAS* mutation in the present study was only 5.0%, much lower than that reported in Caucasians and even lower than that in other Asian countries [18,22]. Since there is a significant association between *KRAS* mutations and smoking behaviors [22,24], the relatively high rate of non-smoking lung cancers in Taiwan might be one reason for the lower *KRAS* mutation rate in the present study. Herein, our results disclosed the higher *EGFR* mutation rate in female and non-smokers and higher *KRAS* mutation rate in male and smokers, which were in line with the results of previous studies [24,25].

*KRAS* mutations in lung adenocarcinoma consist of single amino acid substitutions in hotspots located mostly in codon 12 and rarely in codons 13 and 61 [26,27]. In our study, codon 12 and codon 13 mutations were detected, with much higher mutation rate in codon 12 than in codon 13 (95.7% vs. 4.3%). There is evidence that different *KRAS* mutant proteins have differing clinical significance. In previous study, either G12C or G12V-mutant *KRAS* predicted shorter progression free survival to EGFR-TKI compared to wild type or other *KRAS* mutations [28]. Despite the lack of effective *KRAS*-targeted agents currently, detection of *KRAS* mutations could be considered to apply in clinical practice for its prognostic value [29,30].

The rate of *BRAF* V600E mutation was 0.7% (12/1772) in the present study with eight non-smokers and four smokers. In the study by An et al., *BRAF* mutation rate was 2.3% (7/307) in lung adenocarcinoma, while *BRAF* exon 15 mutation rate was 0.98% (3/307) [31]. The *BRAF* V600E mutation rate was similar to that in our study. Our results showed that there was no significant association of *BRAF* V600E mutation with patients’ age, gender, smoking status or tumor stage. This differs from a recent meta-analysis by Chen et al. [32], showing that *BRAF* V600E mutation was significantly correlated with female gender and non-smoking history. Because different cohorts of patients and detection methods potentially confound the screening of uncommon mutations, further studies are needed to clarify the nature of *BRAF* mutations. Although *BRAF* mutations are uncommon, they represent an important therapeutic target due to the availability of individualized therapies in clinical use for melanoma and the promising results reported in ongoing clinical trials for NSCLC patients [33].

*HER2* mutations consist of in-frame insertions in exon 20, especially HER2YVMA mutant, leading to constitutive activation of the receptor and downstream *AKT* and *MEK* pathways [34]. *HER2* mutations meet the definition of genetic driver and the concept of transforming property of such a genetic alteration has been proved in preclinical models [35]. In the study by Mazieres et al., *HER2* mutation was identified in 65 (1.7%) of 3800 patients tested with a high proportion of women (69%) and never-smokers (52.3%) [36]. In our study, similar *HER2* mutation rate (2.0%) was found with a higher mutation rate in patients with advanced stage diseases. As several agents might be active in *HER2*-mutant lung adenocarcinoma, it is important to screen for *HER2* mutations for the potential efficacy of *HER2*-targeted medications.

*ALK* rearrangement has been demonstrated to be a potent oncogenic driver and a promising therapeutic target in NSCLC. The rate of *ALK* rearrangements ranged from 3 to 7% of unselected patients with NSCLC [37–39]. Similar to *EGFR* mutations, *ALK* rearrangements are associated with distinct clinical features, including younger age at diagnosis, never of light smokers and adenocarcinoma histology. Phase 3 study of crizotinib in previously treated patients with *ALK*-rearranged advanced NSCLC showed an ORR of 65% and a PFS of 7.7 months, which was significantly superior to the results with standard chemotherapy [8].

FISH analysis is the only approved diagnostic test to detect break-apart signals in *ALK* rearrangement. However, the apparatuses required for FISH analysis may not be available in all diagnostic laboratories. IHC can be an alternative to FISH and using the highly sensitive detection methods in combination with high affinity antibodies, IHC can effectively detect *ALK* fusion protein in lung adenocarcinoma with high sensitivity and specificity [40,41]. Automated IHC assay system devised by Ventana was used in this study. Ying et al. assessed 196 lung adenocarcinomas using Ventana IHC, FISH, Cell Signaling Technology (CST) IHC and RT—PCR and the results showed that 65 (33%), 63 (32%), 70 (36%) and 69 (35%) cases were *ALK* positive, respectively [13]. The sensitivity and specificity of Ventana IHC were 100% and 98%. The automated Ventana IHC system is desirable to be considered as a guide to prescribe ALK inhibitors treatment, as IHC is a routine methodology in most pathology laboratories to detect a protein of interest. In the case of the availability of tumor tissues, our results may be slightly skewed due to the small sample size tested for *ALK* rearrangements.

In the present study, around 35% of lung adenocarcinoma patients did not have detectable oncogenic drivers. The comprehensive molecular profiling of 230 resected lung adenocarcinomas reported recently by Cancer Genome Atlas Research Network also showed that 24.4% of patients had no detectable driver oncogenic mutations at initial identification of candidate driver genes [17].

In summary, our study demonstrates that lung adenocarcinoma defined by specific driver gene alterations could be considered as different diseases. The identification of driver mutations has heralded a new era of targeted therapy in lung adenocarcinoma with currently available and potentially targetable genetic aberrations.

## Supporting Information

S1 Table The PCR primers and probes used to genetic analysis in the present study. (PDF)
