# Supplementary material for: Identification of Five Driver Gene Mutations in Patients with Treatment-Naïve Lung Adenocarcinoma in Taiwan
Source: PLoS One. 2015 Mar 19;10(3):e0120852. doi: 10.1371/journal.pone.0120852 (PMC4366385; doi:10.1371/journal.pone.0120852)
Supplement: S1 Table — (PDF) [file pone.0120852.s001.pdf]

Table S1. Sequence of PCR Primers and MassARRAY Probes

Probe ID	Targeted Mutation Site	Sequance (5'->3')
PCR primer		
BRAF_PCRF	BRAF c.1799T>A, p.(Val600Glu)	ACGTTGGATGTCTTCATGAAGACCTCACAG
BRAF_PCRR	BRAF c.1799T>A, p.(Val600Glu)	ACGTTGGATGTTCAAACTGATGGGACCCAC
KRAS_PCRF	KRAS c.34G>A, p.(Gly12Ser)<br>KRAS c.34G>C, p.(Gly12Arg)<br>KRAS c.34G>T, p.(Gly12Cys)<br>KRAS c.35G>A, p.(Gly12Asp)<br>KRAS c.35G>C, p.(Gly12Ala)<br>KRAS c.35G>T, p.(Gly12Val)<br>KRAS c.37G>A, p.(Gly13Ser)<br>KRAS c.37G>T, p.(Gly13Cys)<br>KRAS c.37G>C, p.(Gly13Arg)<br>KRAS c.38G>A, p.(Gly13Asp)<br>KRAS c.38G>T, p.(Gly13Ala)<br>KRAS c.38G>C, p.(Gly13Val)	ACGTTGGATGTAGCTGTATCGTCAAGGCAC
KRAS_PCRR	KRAS c.34G>A, p.(Gly12Ser)<br>KRAS c.34G>C, p.(Gly12Arg)<br>KRAS c.34G>T, p.(Gly12Cys)<br>KRAS c.35G>A, p.(Gly12Asp)<br>KRAS c.35G>C, p.(Gly12Ala)<br>KRAS c.35G>T, p.(Gly12Val)<br>KRAS c.37G>A, p.(Gly13Ser)<br>KRAS c.37G>T, p.(Gly13Cys)<br>KRAS c.37G>C, p.(Gly13Arg)<br>KRAS c.38G>A, p.(Gly13Asp)<br>KRAS c.38G>T, p.(Gly13Ala)<br>KRAS c.38G>C, p.(Gly13Val)	ACGTTGGATGACTTGTGGTAGTTGGAGCTG
HER2_PCRF	HER2 c.2325_2326>TATACGTGATGGCG,<br>p.(A775_G776insYVMA)	ACGTTGGATGAGAAGGCGGGAGACATATGG
HER2_PCRR	HER2 c.2325_2326>TATACGTGATGGCG,<br>p.(A775_G776insYVMA)	ACGTTGGATGTACCCTTGTCCCCAGGAAG
EGFR_ex18_PCRF	EGFR c.2126A>C, p.(Glu709Ala)<br>EGFR c.2126A>G, p.(Glu709Gly)<br>EGFR c.2126A>T, p.(Glu709Val)<br>EGFR c.2156G>C, p.(Gly719Ala)<br>EGFR c.2156G>A, p.(Gly719Asn)<br>EGFR c.2155G>A, p.(Gly719Ser)<br>EGFR c.2155G>T, p.(Gly719Cys)	ACGTTGGATGTTATACACCGTGCCGAACGC
EGFR_ex18_PCRR	EGFR c.2126A>C, p.(Glu709Ala)<br>EGFR c.2126A>G, p.(Glu709Gly)<br>EGFR c.2126A>T, p.(Glu709Val)<br>EGFR c.2156G>C, p.(Gly719Ala)<br>EGFR c.2156G>A, p.(Gly719Asn)<br>EGFR c.2155G>A, p.(Gly719Ser)<br>EGFR c.2155G>T, p.(Gly719Cys)	ACGTTGGATGACCAAGCTCTCTTGAGGATC
EGFR_ex19_PCRF	EGFR c.2235_2249del15, p.(E746-A750del)<br>EGFR c.2236_2250del15, p.(E746-A750del)<br>EGFR c.2237_2251del15, p.(E746-T751>A)<br>EGFR c.2237_2244>T, p.(E746-S752>V)<br>EGFR c.2239_2248>C, p.(L747-E750>P)<br>EGFR c.2239_2251>C, p.(L747-T751>P)<br>EGFR c.2239_2256del18, p.(L747-S752del)<br>EGFR c.2240_2254del15, p.L747-T751del)<br>EGFR c.2240_2257del19, p.(L747-P753>S)<br>EGFR c.2235_2252>AAT, p.(E746-T751>I)<br>EGFR c.2236_2253del18, p.(E746-T751del)<br>EGFR c.2237_2254del18, p.(E746-S752>A)<br>EGFR c.2238_2255del18, p.(E746-S752>D)<br>EGFR c.2238_2248>GC, p.(L747-A750>P)<br>EGFR c.2238_2252>GCA, p.(L747-T751>Q)<br>EGFR c.2239_2247del9, p.(L747-E749del)<br>EGFR c.2239_2253del15, p.(L747-T751del)<br>EGFR c.2239_2258>CA, p.(L747-P753>Q)<br>EGFR c.2240_2251del12, p.(L747-T751>S)	ACGTTGGATGAGCAGAAACTCACATCGAGG
EGFR_ex19_PCRR	EGFR c.2235_2249del15, p.(E746-A750del)<br>EGFR c.2236_2250del15, p.(E746-A750del)<br>EGFR c.2237_2251del15, p.(E746-T751>A)<br>EGFR c.2237_2244>T, p.(E746-S752>V)<br>EGFR c.2239_2248>C, p.(L747-E750>P)<br>EGFR c.2239_2251>C, p.(L747-T751>P)<br>EGFR c.2239_2256del18, p.(L747-S752del)<br>EGFR c.2240_2254del15, p.L747-T751del)<br>EGFR c.2240_2257del19, p.(L747-P753>S)<br>EGFR c.2235_2252>AAT, p.(E746-T751>I)<br>EGFR c.2236_2253del18, p.(E746-T751del)<br>EGFR c.2237_2254del18, p.(E746-S752>A)<br>EGFR c.2238_2255del18, p.(E746-S752>D)<br>EGFR c.2238_2248>GC, p.(L747-A750>P)<br>EGFR c.2238_2252>GCA, p.(L747-T751>Q)	ACGTTGGATGGATCCCAGAAGGTGAGAAAG

	EGFR c.2239_2247del9, p.(L747-E749del)<br>EGFR c.2239_2253del15, p.(L747-T751del)<br>EGFR c.2239_2258>CA, p.(L747-P753>Q)<br>EGFR c.2240_2251del12, p.(L747-T751>S)	
EGFR_ex20_PCRF	EGFR c.2303G>T, p.(Ser768Ile)<br>EGFR c.2369C>T, p.(Thr790Met)	ACGTTGGATGTGTTCCCGGACATAGTCCAG
EGFR_ex20_PCRR	EGFR c.2303G>T, p.(Ser768Ile)<br>EGFR c.2369C>T, p.(Thr790Met)	ACGTTGGATGATCTGCCTCACCTCCACCGT
EGFR_ex21_PCRF	EGFR c.2573T>G, p.(Leu858Arg)<br>EGFR c.2573T>A, p.(Ler858Gln)<br>c.2582T>A, p.(Leu861Gln)	EGFR ACGTTGGATGCCTCCTTCTGCATGGTATTC
EGFR_ex21_PCRR	EGFR c.2573T>G, p.(Leu858Arg)<br>EGFR c.2573T>A, p.(Ler858Gln)<br>c.2582T>A, p.(Leu861Gln)	EGFR ACGTTGGATGGCAGCATGTCAAGATCACAG
Probe		
BRAF_1799	BRAF c.1799T>A, p.(Val600Glu)	CCACTCCATCGAGATTTC
KRAS_215	KRAS c.34G>A, p.(Gly12Ser)<br>KRAS c.34G>C, p.(Gly12Arg)<br>KRAS c.34G>T, p.(Gly12Cys)	TAAACTTGTGGTAGTTGGAGCT
KRAS_216	KRAS c.35G>A, p.(Gly12Asp)<br>KRAS c.35G>C, p.(Gly12Ala)<br>KRAS c.35G>T, p.(Gly12Val)	AGGCACTCTTGCCTACGCCA
KRAS_218	KRAS c.37G>A, p.(Gly13Ser)<br>KRAS c.37G>T, p.(Gly13Cys)<br>KRAS c.37G>C, p.(Gly13Arg)	TCGTCAAGGCACTCTTGCCTACGC
KRAS_219	KRAS c.38G>A, p.(Gly13Asp)<br>KRAS c.38G>T, p.(Gly13Ala)<br>KRAS c.38G>C, p.(Gly13Val)	GCACTCTTGCCTACG
HER2_2325	HER2 c.2325_2326>TATACGTGATGGCG,<br>p.(A775_G776insYVMA)	AGCATACGTGATGGC
HER2_2326	HER2 c.2325_2326>TATACGTGATGGCG,<br>p.(A775_G776insYVMA)	AAGCATACGTGATGGCT
EGFR_2126	EGFR c.2126A>C, p.(Glu709Ala)<br>EGFR c.2126A>G, p.(Glu709Gly)<br>EGFR c.2126A>T, p.(Glu709Val)	CAAGCTCTCTTGAGGATCTTGAAGG
EGFR_2155	EGFR c.2155G>A, p.(Gly719Ser)<br>EGFR c.2155G>T, p.(Gly719Cys)	AATTCAAAAAGATCAAAGTGCTG
EGFR_2156	EGFR c.2156G>C, p.(Gly719Ala)<br>EGFR c.2156G>A, p.(Gly719Asn)	GTGCCGAACGCACCGGAG
EGFR_del1	EGFR c.2235_2249del15, p.(E746-A750del)	ATTCCCGTCGCTATCAA
EGFR_del2	EGFR c.2236_2250del15, p.(E746-A750del)	ATTCCCGTCGCTATCAAG
EGFR_del3/4	EGFR c.2237_2251del15, p.(E746-T751>A)<br>EGFR c.2237_2244>T, p.(E746-S752>V)	ATTCCCGTCGCTATCAAGG
EGFR_del5	EGFR c.2239_2248>C, p.(L747-E750>P)	GGCTTTCGGAGATGTTG
EGFR_del6	EGFR c.2239_2251>C, p.(L747-T751>P)	TTGTTGGCTTTCGGAGATG
EGFR_del7	EGFR c.2239_2256del18, p.(L747-S752del)	CCTTGTTGGCTTTCGG
EGFR_del8	EGFR c.2240_2254del15, p.L747-T751del)	TCCTTGTTGGCTTTCGGAGAT
EGFR_del9	EGFR c.2240_2257del19, p.(L747-P753>S)	TCGAGGATTTCCTTGTTGGCTTTCG
EGFR_del13	EGFR c.2238_2255del18, p.(E746-S752>D)	AATTCCCGTCGCTATCAAGGA
EGFR_del16	EGFR c.2239_2247del9, p.(L747-E749del)	AATTCCCGTCGCTATCAAGGAA
EGFR_del17	EGFR c.2239_2253del15, p.(L747-T751del)	AAATTCCCGTCGCTATCAAGGAAT
EGFR_2303	EGFR c.2303G>T, p.(Ser768Ile)	GGCGGCACACGTGGGGGTTGTCCACG
EGFR_2369	EGFR c.2369C>T, p.(Thr790Met)	TGCCTCACCTCCACCGTGCAGCTCATCA
EGFR_2573	EGFR c.2573T>G, p.(Leu858Arg)<br>EGFR c.2573T>A, p.(Ler858Gln)	AAGATCACAGATTTTGGGC
EGFR_2582	EGFR c.2582T>A, p.(Leu861Gln)	TTCTTTCTCTTCCGCACCCAGC
